# The Phospholipase Activity of Ammodytoxin, a Prototype Snake Venom β-Neurotoxin, Is Not Obligatory for Cell Internalisation and Translocation to Mitochondria

**DOI:** 10.3390/toxins14060375

**Published:** 2022-05-28

**Authors:** Adrijan Ivanušec, Jernej Šribar, Peter Veranič, Igor Križaj

**Affiliations:** 1 Department of Molecular and Biomedical Sciences, Jožef Stefan Institute, SI-1000 Ljubljana, Slovenia; adrijan.ivanusec@ijs.si (A.I.); jernej.sribar@ijs.si (J.Š.); 2 Doctoral School, Faculty of Medicine, University of Ljubljana, SI-1000 Ljubljana, Slovenia; 3 Faculty of Medicine, Institute of Cell Biology, University of Ljubljana, SI-1000 Ljubljana, Slovenia

**Keywords:** β-neurotoxicity, secreted phospholipase A_2_, ammodytoxin, snake venom, *Vipera ammodytes ammodytes*, transmission electron microscopy, localisation, translocation

## Abstract

β-Neurotoxins are secreted phospholipase A_2_ molecules that inhibit transmission in neuromuscular synapses by poisoning the motor neurons. These toxins specifically and rapidly internalise into the nerve endings of motor neurons. Ammodytoxin (Atx) is a prototype β-neurotoxin from the venom of the nose-horned viper (*Vipera ammodytes ammodytes*). Here, we studied the relevance of the enzymatic activity of Atx in cell internalisation and subsequent intracellular movement using transmission electron microscopy (TEM). We prepared a recombinant, enzymatically inactive mutant of Atx, Atx(D49S), labelled with gold nanoparticles (GNP), and incubated this with PC12 cells, to analyse its localisation by TEM. Atx(D49S)-GNP internalised into the cells. Inside the cells, Atx(D49S)-GNP was detected in different vesicle-like structures, cytosol, endoplasmic reticulum and mitochondria, where it was spotted in the intermembrane space and matrix. Co-localization of fluorescently labelled Atx(D49S) with mitochondria in PC12 cells by confocal fluorescence microscopy confirmed the reliability of results generated using Atx(D49S)-GNP and TEM and allowed us to conclude that the phospholipase activity of Atx is not obligatory for its cell internalisation and translocation into the mitochondrial intermembrane space and matrix.

## 1. Introduction

β-Neurotoxins (β-NTXs) are presynaptically toxic, secreted phospholipase A_2_ (sPLA_2_s; EC 3.1.1.4) enzymes that release free fatty acids and lysophospholipids from glycerophospholipids by cleaving the ester bond at the *sn*-2 position [1]. β-NTXs can be found in the venoms of Elapidae and Viperidae snakes. Those from elapid venoms belong to Group IA sPLA_2_s, while those from viperid venoms belong to Group IIA sPLA_2_s. Neurotoxic sPLA_2_s poison neuro-muscular junctions by inducing blockade of exocytosis of neurotransmitter-filled synaptic vesicles, reduction in the number of synaptic vesicles in the nerve endings, swelling and degeneration of the mitochondria accompanied by the release of alarmins, and destruction of the nerve terminals [2,3,4]. Altogether, this leads to flaccid paralysis of the victim.

Ammodytoxin (Atx) is a β-NTX from the venom of the nose-horned viper (*Vipera ammodytes ammodytes*) that has been shown to cross the plasma membrane (PM) of mouse motor nerve terminals, to reach the cytosol and mitochondria [5]. Internalisation of Atx and other β-NTXs into cells has also been shown using neuronal cell lines in culture [6,7,8,9]. In addition, Atx was shown to bind several intracellular proteins with high affinity, such as calmodulin, 14-3-3 proteins, protein disulphide isomerase, and cytochrome c oxidase subunit II (CCOX-II) [10]. Nevertheless, the molecular mechanisms of its internalisation into cells and its intracellular trafficking are far from clear. Several cell-internalisation pathways have been suggested, such as through recycling of synaptic vesicle or by retrograde transport via the endoplasmic reticulum after receptor-mediated endocytosis from the PM [9,11].

It is generally accepted that the phospholipase activity of β-NTXs is obligatory for the full expression of their neurotoxicity [4]. In particular, their enzymatic activity appears to be crucial at the appropriate locations within cells. Nevertheless, the phospholipase activity alone is not sufficient for the β-neurotoxic effects. β-neurotoxicity is a multistep process that is composed of steps in which the binding of a β-NTX to specific cellular targets is important, and steps in which the phospholipase activity of a β-NTX takes part. The intracellular actions of β-NTXs appear to be crucial determinants for their β-neurotoxicity, so a precise description of the cellular trafficking of β-NTXs is a prerequisite to the design of effective protection strategies, or for the development of these toxins as molecular tools. 

To this end, we addressed here the question of whether the phospholipase activity of Atx, a prototype β-NTX, is essential for its cell internalisation and translocation inside the cell. We used a new high-resolution approach to shed more light on Atx internalisation and cellular localisation: transmission electron microscopy (TEM) using a gold nanoparticle (GNP)-derivatised enzymatically inactive mutant of Atx as a tracer.

## 2. Results

### 2.1. Biochemical Characterisation of Recombinant Atx(D49S)

The recombinant enzymatically inactive mutant of Atx, Atx(D49S), was produced in *Escherichia* *coli* in the form of inactive inclusion bodies and was refolded in vitro. We initially confirmed that the recombinant protein was homogenous, without any post-translational modifications, and that it was correctly folded and functional, using analytical methods (i.e., SDS-PAGE, N-terminal protein sequencing, mass spectrometry, affinity-binding studies). Compared to the native toxin, Atx(D49S) showed negligible enzymatic activity on PyrPG vesicles as substrate (Supplementary Materials Figure S1).

We then conjugated recombinant Atx(D49S) and 5-nm gold nanoparticles (GNP) to obtain Atx(D49S)-GNP for the TEM studies. Furthermore, Atx(D49S) was conjugated with the ^546^Alexa dye for the fluorescence studies. This fluorescent derivative of Atx(D49S), ^546^Alexa-Atx(D49S), was characterised as described above for Atx(D49S). Its binding to calmodulin did not differ from that of Atx(D49S).

### 2.2. Localisation of Atx(D49S)-GNP in PC12 Cells

**Figure 1:**
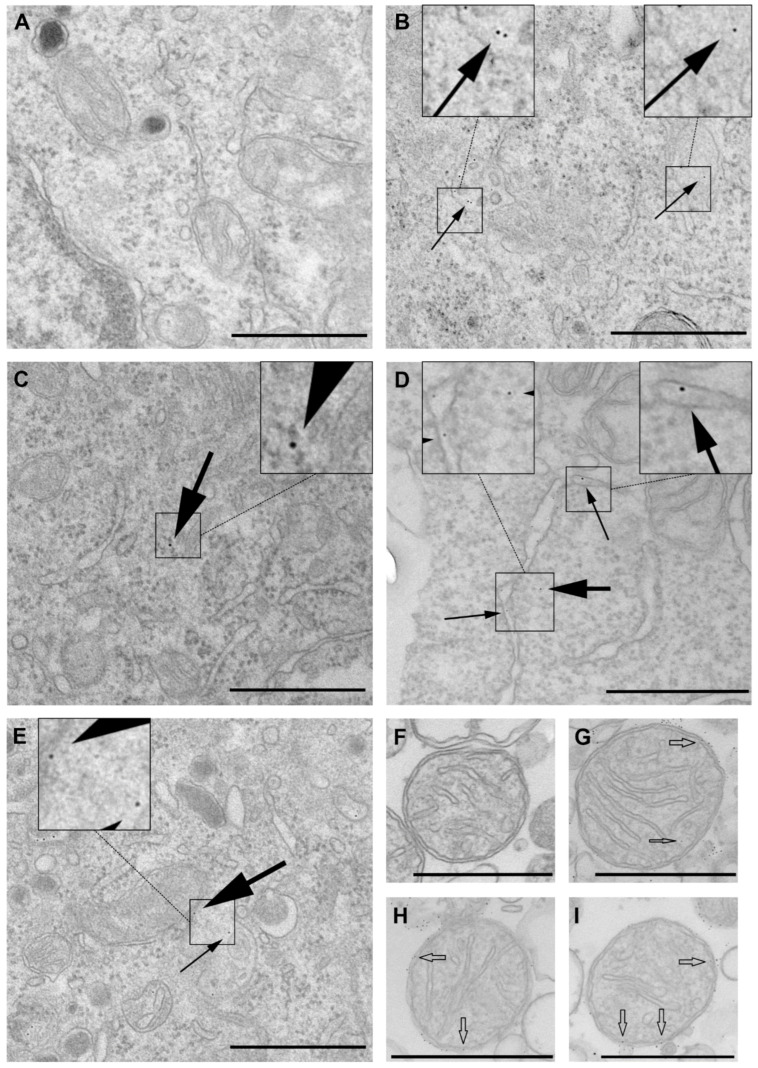
Distribution of the enzymatically inactive mutant of ammodytoxin conjugated to gold nanoparticles, Atx(D49S)-GNP, in PC12 cells and in their isolated mitochondria using transmission electron microscopy (TEM). (**A**–**E**) Representative TEM images of PC12 cells incubated without (**A**) and with Atx(D49S)-GNP (**B**–**E**) for 2 h. Electron-dense particles were concentrated in different vesicular structures ((**B**,**E**) small black arrows), in the cytosol ((**C**,**D**) large black arrows) and in the endoplasmic reticulum ((**D**) small black arrows), or were closely associated with the mitochondria ((**E**) large black arrow). For easier perception, the areas with golden particles are zoomed in (insets). No similar signals were seen for the control cells (**A**), indicating that the electron-dense particles corresponded to Atx(D49S)-GNP. No morphological changes were apparent in control (**A**) and Atx(D49S)-GNP–exposed cells (**B**–**E**). (**F**–**I**) Representative TEM images of mitochondria isolated from PC12 cells and incubated without (**F**) and with Atx(D49S)-GNP (**G**–**I**) for 1 h. Atx(D49S)-GNP was mainly associated with the outer mitochondrial membrane, with some also seen in the mitochondrial intermembrane space ((**G**–**I**) wide empty arrows) and in the matrix ((**G**), narrow empty arrow) of apparently intact mitochondria. No morphological changes were apparent in the control and Atx(D49S)-GNP-exposed mitochondria. Scale bars, 600 nm.

To gain insight into the intracellular pathways of Atx and the role of its enzymatic activity in cellular translocation, Atx(D49S)-GNP was incubated with PC12 cells for 2 h and tracked using TEM. When counterstaining of ultrathin sections was not used, no signal for GNP was seen in the sections, therefore we interpreted the electron-dense particles as Atx(D49S)-GNP. No morphological changes were detected in the Atx(D49S)-GNP–exposed cells compared to control cells (Figure 1A). Atx(D49S)-GNP was internalised into the PC12 cells and localised in different vesicular structures, which resembled early endosomes, multivesicular bodies and late endosomes (Figure 1B,E). To some extent, Atx(D49S)-GNP was also seen in the endoplasmic reticulum and cytosol, and was closely associated to mitochondria (Figure 1C–E). When lysozyme-GNP was used instead of Atx(D49S)-GNP, the signal was observed only in endocytotic vesicles, but not in the cytosol or endoplasmic reticulum (Supplementary Materials Figure S2).

To determine whether Atx(D49S) can translocate into the mitochondria independent of non-mitochondrial factors, mitochondria were isolated from PC12 cells and incubated with Atx(D49S)-GNP, and the localisation of Atx(D49S)-GNP was analysed using TEM. Here, Atx(D49S)-GNP associated mainly with the outer mitochondrial membrane; however, some specific signals were also seen in the mitochondrial intermembrane space and the matrix of apparently intact mitochondria (Figure 1G–I). No morphological changes were seen for these Atx(D49S)-GNP–treated mitochondria compared to the control mitochondria (Figure 1F).

### 2.3. Localisation of ^546^Alexa-Atx(D49S) in PC12 Cells

**Figure 2:**
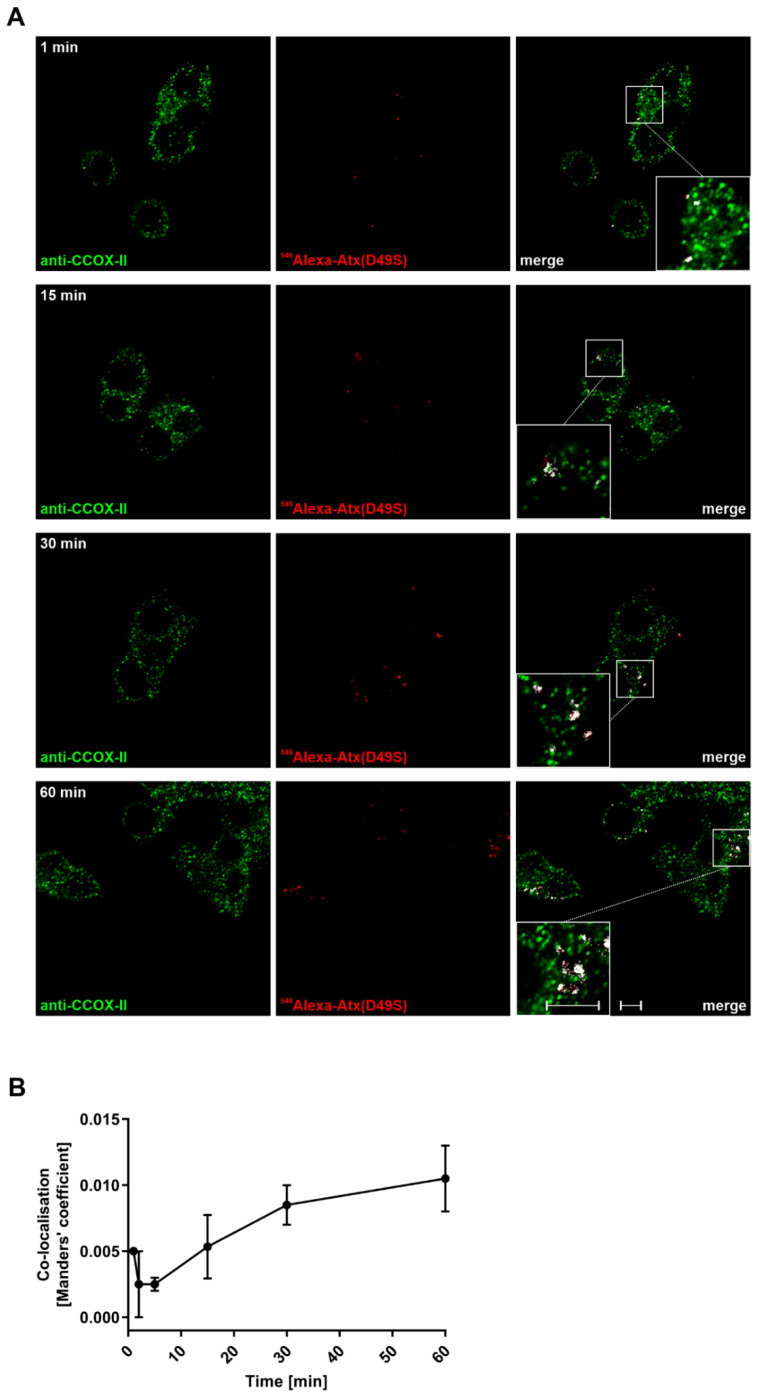
Atx(D49S) co-localises with mitochondria in PC12 cells. PC12 cells were incubated with 100 nM ^546^Alexa-Atx(D49S) for 1, 2, 5, 15, 30 and 60 min. The cells were fixed, stained with anti-CCOX-II antibodies (i.e., mitochondria) and analysed. (**A**) Representative confocal fluorescence microscopy images showing ^546^Alexa-Atx(D49S) (red) and anti-CCOX-II antibodies (green); co-localisation in merged images is shown in white. Insets: Magnified views of the boxed areas of the cells. Scale bars, 5 µm (apply to all images). (**B**) Time course of co-localisation of ^546^Alexa-Atx(D49S) and anti-CCOX-II antibodies from stacks of images acquired as in (**A**). The extent of co-localisation of Atx(D49S) and mitochondria in PC12 cells was calculated and expressed in terms of the Manders’ coefficient, with the data presented as a function of time. The co-localisation signals became stronger at incubation times > 30 min. Data are means ± standard error of the mean, calculated from at least two sets of images for each time point.

To determine the reliability of the data generated with Atx(D49S)-GNP using TEM, an alternative localisation procedure was used, with fluorescently labelled Atx(D49S) and confocal microscopy. PC12 cells were incubated with ^546^Alexa-Atx(D49S) for 1, 2, 5, 15, 30 and 60 min, fixed, and then immunostained with anti-CCOX-II antibodies and ^488^Alexa-conjugated secondary antibodies. CCOX-II is the specific receptor for Atx in the inner mitochondrial membrane [12]. The distribution of the signals for ^546^Alexa-Atx(D49S) and CCOX-II in the cells was obtained by acquiring multiple optical sections for each sample under confocal fluorescence microscopy (Figure 2A). Stacks of images of each sample were quantified and the degree of co-localisation of these signals was determined using the ZEN software (ZEISS, Jena, Germany). To determine the threshold signal for each channel, the autofluorescence and background signals were eliminated under control conditions, in which either ^546^Alexa-Atx(D49S) or the ^488^Alexa-conjugated secondary antibodies were omitted. Figure 2B shows the results in terms of the Manders’ coefficient. Co-localisation of ^546^Alexa-Atx(D49S) and the CCOX-II signals were significant at incubation times > 30 min.

Thus, we demonstrated that both these analytic techniques of TEM and confocal fluorescence microscopy indicated the same localisation of Atx(D49S) in PC12 cells. We can conclude that the phospholipase activity of Atx is not obligatory for its internalisation into PC12 cells and its subsequent translocation to the mitochondria. The high resolving power of TEM allowed direct observation of Atx(D49S)-GNP in the mitochondrial intermembrane space and the matrix.

## 3. Discussion

Despite extensive studies, the molecular mechanisms of action of β-NTXs are still not fully understood. One of the main unresolved questions remains how β-NTXs enter the nerve cells, translocate into their cytosol, and reach the intracellular organelles, such as the mitochondria. At least two cell-internalisation pathways for β-NTXs have been proposed: via synaptic vesicles during their recycling from the PM after exocytosis, and by receptor-mediated endocytosis using the retrograde protein transport mechanism [4]. Several specific binding proteins for β-NTXs on the PM of nerve and other cells have been characterised (for reviews, see [10,13]); however, none of these have been firmly associated with the process of β-neurotoxicity to date. As the intracellular actions of β-NTXs appear to be essentially responsible for β-neurotoxicity, we addressed here the important issue of the significance of the enzymatic activity of β-NTXs for their trafficking into and within cells. 

To tackle this question, we used Atx, a prototype β-NTX from nose-horned viper venom. In the first step, we created a mutant Atx without enzymatic activity, Atx(D49S). Atx(D49S) includes a single amino acid replacement to preserve the three-dimensional structure of Atx. The key amino acid residues for the enzymatic activity of sPLA_2_s are those of the catalytic network: His48, Asp49, Tyr52 and Asp99. By analogy with ammodytin L, an enzymatically inactive Atx homologue from the same snake venom [14], we mutated Asp49 to Ser to produce this enzymatically inactive Atx(D49S). This generated a correctly folded sPLA_2_ molecule that retained all of the characteristics of the native Atx, except for its enzymatic activity.

PC12 cells have properties similar to those of neurons [15], the natural targets of β-NTXs. PC12 cells have been shown to represent a suitable cell model to study cell internalisation and intracellular pathways of β-NTXs. We demonstrated previously that Atx can enter the cytosol of PC12 cells, to interact with several cytosolic proteins, an endoplasmic-reticulum-residing protein, and a protein in the inner mitochondrial membrane of PC12 cells [9,12]. The choice to use PC12 cells to study the role of the phospholipase activity in cellular trafficking of Atx was therefore logical. 

Due to the higher resolution in comparison to confocal laser fluorescence microscopy, this study used TEM with GNP-conjugated Atx(D49S) as a new experimental approach to analyse the role of the enzymatic activity in cellular trafficking of β-NTXs. Analysis of the electron micrographs indicated that Atx(D49S)-GNP was internalised into the PC12 cells. Electron-dense spots were detected within vesicular structures apparently of endocytic origin, which is consistent with previous reports on β-NTX internalisation into cells [5,7,8,16,17]. Although with lower frequency, Atx(D49S)-GNP signals were also seen in the cytosol, endoplasmic reticulum and mitochondria. Similar observations were reported for the enzymatically active GNP derivative of Atx(N79C) in motor neuron-like cells [7] and in mouse motor nerve terminals [5]. This indicated that the enzymatic activity of Atx is not essential for translocation of the sPLA_2_ molecule into the target cells, which thus defines further specific interactions of Atx with the target cell as vitally important for translocation and cellular trafficking of this β-NTX. 

The attachment of a large label (GNP: diameter, 5 nm) to an sPLA_2_ molecule of similar dimensions [18] might, however, interfere with the interactions of the sPLA_2_ with binding proteins in the cell, and thus affect its trafficking. We therefore determined the distribution of Atx(D49S) labelled with a small fluorescent label (^546^Alexa) in PC12 cells by confocal fluorescence microscopy to compare this with the distribution of Atx(D49S)-GNP. In these PC12 cells, the distribution of ^546^Alexa-Atx(D49S) was very similar to that of Atx(D49S)-GNP, so we can conclude that monitoring of Atx(D49S)-GNP by TEM represents a relevant high-resolution approach to the analysis of the cellular trafficking of Atx.

The distribution of ^546^Alexa-Atx(D49S) in PC12 cells was also the same as the distribution of the ^546^Alexa-tagged wild-type Atx (i.e., enzymatically active) [12]. This thus further strengthens the conclusion stemming from this TEM study: the enzymatic activity of Atx is not a prerequisite for its internalisation and intracellular trafficking. 

The high resolution of TEM also allowed us to examine the distribution of Atx(D49S)-GNP inside the mitochondria. Following incubation of isolated PC12 mitochondria with Atx(D49S)-GNP, the specific signal was associated with the outer mitochondrial membrane, the mitochondrial intermembrane space and the matrix. This is consistent with the localisation of CCOX, the specific receptor for Atx and a component of the respiratory chain in the inner membrane of the mitochondria [12]. Rat GIIA sPLA_2_ is an orthologue of Atx, and it has also been detected in mitochondria [19]. However, how either endogenous or exogenous sPLA_2_s enter mitochondria is currently not clear, as these proteins do not have any known mitochondrion-targeting sequences. They might follow the Mia40 route, as it has been shown that some proteins without mitochondrion-targeting determinants can enter mitochondria via this mitochondrial receptor [20]. 

Characteristic cell damage is a known indicator of β-neurotoxicity, which includes mitochondrial degeneration [21,22]. However, exposure of PC12 cells or their isolated mitochondria to Atx(D49S)-GNP here did not induce any morphological changes. This indicates that Atx(D49S)-GNP was not toxic to the cells or to mitochondria *per se*. This is consistent with the view that the enzymatic activity of β-NTXs is crucial for the expression of their toxicity [4]. 

To summarise, here we have demonstrated that the use of Atx(D49S)-GNP and TEM represents a relevant approach for high-resolution studies of cell internalisation and intracellular trafficking of sPLA_2_s. Using this approach, we demonstrated that the enzymatic activity of Atx is not essential for its passage through the PM to reach the cytosol, nor for its entry into mitochondria. Detailed analysis of mitochondrial distribution of Atx(D49S)-GNP revealed that this probe entered the mitochondrial intermembrane space and the matrix. It is very likely that Atx and other β-NTXs move within cells along the pathways used by their mammalian orthologues, and thus they represent excellent tools for the characterisation of these pathways.

## 4. Materials and Methods

### 4.1. Materials

The expression plasmid for the production of recombinant Atx(D49S) was constructed by Dr. Jernej Oberčkal, Jožef Stefan Institute (Ljubljana, Slovenia). Mouse anti-CCOX-II antibodies and goat anti-mouse IgG labelled with Alexa Fluor 488 antibodies were from Invitrogen (Carlsbad, CA, USA). All other chemicals were of analytical, sequencing or mass spectrometry grades.

### 4.2. Production and Purification of the Recombinant Enzymatically Inactive Mutant of Atx

Atx(D49S) is a mutant of Atx that lacks the phospholipase enzymatic activity due to the point mutation in the active site. It was produced by in vitro refolding of the inclusion body protein produced by expression in *E. coli* BL21 (DE3). An expression plasmid (based on pT7-7; [23]) with a cDNA insert that coded for the mature Atx(D49S) was used for expression by auto-induction using a method adapted from Studier (2005) [24]. The expression plasmid was transformed into competent *E. coli* BL21 (DE3) cells, and the cells were grown overnight on Luria-Bertani agar plates supplemented with ampicillin. The next day, the cells were inoculated into Luria-Bertani medium and grown to an optical density at 600 nm (OD_600_) of 0.7. The bacterial cultures were then diluted 100-fold in the auto-induction medium (1.5% (*v*/*v*) glycerol, 0.05% (*m*/*v*) glucose, 0.2% (*m*/*v*) lactose, 0.01 mM FeSO_4_, 2 mM MgCl_2_, 0.01 mM CaCl_2_, 0.05% (*m*/*v*) NH_4_Cl, 0.05% (*m*/*v*) NaCl, 0.3% (*m*/*v*) KH_2_PO_4_, 0.6% (*m*/*v*) Na_2_HPO_4_, 0.01 mg/mL thiamine, in Luria-Bertani medium). The cells were shaken at 230 rpm in 250 mL auto-induction medium in a 2-L flask for 2 h at 37 °C, and then overnight at 25 °C. The next day, when the OD_600_ of the expression mixture remained constant for at least 1 h, the cells were pelleted by centrifugation at 5000× *g* for 20 min at 4 °C. The pellets were frozen at −80 °C until use.

Inclusion bodies and the sulfonated protein were isolated by a method adapted from Valentin et al. (1999) [25]. The bacterial pellets were resuspended for 10 min on ice in 20 mM Tris-HCl, pH 7.5, containing 20% (*m*/*v*) sucrose, 5 mM EDTA, 1 mM dithiothreitol and protease inhibitors, and centrifuged at 11,000× *g* for 10 min at 4 °C. The pellets were resuspended in ice-cold lysis buffer (1 mM dithiothreitol, 10 mM MgSO_4_, protease inhibitors) and the mixture was centrifuged at 11,000× *g* for 10 min at 4 °C. The pellets were resuspended in ice-cold phosphate-buffered saline (PBS) containing 1 mM dithiothreitol and protease inhibitors. The mixture was then sonicated (UP200S ultrasonic processor; Hielscher, Germany) for 2 min with a 50/50 cycle at 80% maximum amplitude, and then centrifuged at 14,000× *g* for 20 min at 4 °C. The resulting pellets were washed twice with ice-cold PBS containing 25% (*m*/*v*) sucrose, 1% (*m*/*v*) Triton X-100, and protease inhibitors, followed by centrifugation at 14,000× *g* for 20 min at 4 °C. The isolated inclusion bodies were left to dissolve overnight at room temperature in 6 M guanidinium hydrochloride containing 0.3 M Na_2_SO_3_, pH 8.5. The next day, 3.75 mL of the sulfonating reagent 2-nitro-5-thiosulfobenzoate was added to the solution, which was then mixed for 1 h at room temperature. After the sulphonation, 20 mL 100% acetic acid was added, followed by 950 mL ice-cold distilled water, and the protein was left to aggregate overnight at 4 °C. The next day, the protein was pelleted by centrifugation at 6000× *g* for 15 min at 4 °C and washed once with distilled water. The resulting pellets were dissolved in 6 M guanidinium hydrochloride, pH 8.3, and the denatured protein was stored at −20 °C until use. The denatured protein was refolded in vitro using the method adapted from Snitko et al. (1997) [26]. The denatured protein was added dropwise (1 drop/10 s) into renaturation buffer (25 mM Tris-HCl, pH 7.5, 5 mM CaCl_2_, 5 mM L-cysteine, 0.9 M guanidinium chloride) under vigorous stirring, and stirred additionally for 48 h at 4 °C. Any precipitate was removed by centrifugation at 9000× *g* for 20 min and filtration through 0.45-µm filters. The protein was concentrated by ultrafiltration using an ultrafiltration cell (Amicon) equipped with a membrane (Omega OM010076; Pall Corporation, Port Washington, NY, USA), and purified to homogeneity by two-step reverse-phase HPLC (series 1100; Hewlett Packard, Palo Alto, CA, USA). In the first step, a Zorbax 300SB-C3 column was used (150 mm × 4.6 mm; Agilent, Santa Clara, CA, USA), with equilibration with 5% (*v*/*v*) solvent B (90% (*v*/*v*) acetonitrile, 0.1% (*v*/*v*) trifluoroacetic acid in water) in solvent A (0.1% (*v*/*v*) trifluoroacetic acid in water). In the second step, a Symmetry Shield RP-18 column was used (250 mm × 4.6 mm; Waters, Milford, MA, USA), with equilibration with 30% (*v*/*v*) solvent B in solvent A. Protein was eluted with a linear gradient of 5% to 100% (*v*/*v*) solvent B in solvent A at a flow rate of 2 mL/min in the first step, and of 30% to 100% (*v*/*v*) solvent B in solvent A at a flow rate of 1 mL/min in the second step. The HPLC peaks were collected, vacuum-dried, dissolved in distilled water and stored at −20 °C.

The recombinant protein was analysed by SDS-PAGE in the presence of 150 mM dithiothreitol on 15% (*m*/*v*) polyacrylamide gels. Its N-terminal sequence was determined by Edman degradation on a protein sequencing system, Procise 492A (Applied Biosystems, Foster City, CA, USA). Mass spectrometry was performed on a mass spectrometer with electrospray ionisation Q-TOF Premier (Waters, Milford, MA, USA). Comparison of the binding to calmodulin between recombinant Atx(D49) and the native toxin was performed as described by Šribar et al. (2003) [27], while comparison of their enzymatic activity on PyrPG vesicles was accomplished as described in Oberčkal et al. (2015) [9].

### 4.3. Culturing of PC12 Cells

PC12 cells (ATCC CRL-1721, American Type Culture Collection, Manassas, VI, USA) are a cell line derived from a pheochromocytoma of the rat adrenal medulla, and these were grown at 37 °C under 5% (*v*/*v*) CO_2_ in 10-cm culture plates in F12K growth medium (Kaighn’s modification of Ham’s F-12; Gibco, Waltham, MA, USA) containing 15% (*v*/*v*) horse serum, 2.5% (*v*/*v*) foetal bovine serum, 100 U/mL penicillin and 100 μg/mL streptomycin (i.e., the culture medium). For confocal microscopy, the cells were plated on poly-L-Lys–coated coverslips, and for transmission electron microscopy, on plastic-bottomed cell-culture dishes with four inner rings (Greiner Bio-One, Frickenhausen, Germany).

### 4.4. Isolation of Mitochondria from PC12 Cells

Mitochondria were prepared from PC12 cells using a method adapted from Monni et al. (2000) [28]. After reaching approximately 75% confluence, the cells were dissociated from the cell culture plates by gentle scraping, homogenised by passing them through a needle (five times; size 26G), and centrifuged at 1000× *g* for 15 min. The pellet was resuspended in buffer A (2 mM Hepes, pH 7.4, 150 mM MgCl_2_, 10 mM KCl) in 6-fold the volume of the pellet, and homogenised using a Potter homogeniser (IKA, Germany). Buffer B (1 M sucrose in buffer A) was added to one third of the previous suspension volume, and the nuclear fraction was pelleted by centrifugation at 1000× *g* for 5 min. The supernatant was decanted and centrifuged at 5000× *g* for 10 min. This new supernatant was discarded, and the pellet was resuspended in buffer C (2 mM Hepes, pH 7.4, 150 mM MgCl_2_, 0.25 M sucrose). The suspension was homogenised using a loose Dounce glass homogeniser (10 passages; Wheaton, IL, USA), and centrifuged at 5000× *g* for 10 min. The pellet of the crude mitochondrial synaptosomal fraction obtained was resuspended in buffer D (2 mM Hepes, pH 7.4, 0.25 M sucrose), and the suspension was applied to the top of a discontinuous sucrose gradient of 0.8-mL layers of 1.6 M, 1.4 M, 1.2 M, 1.0 M and 0.8 M sucrose in 2 mM Hepes, pH 7.4. After centrifugation at 120,000× *g* for 60 min, the mitochondrial fraction was obtained from the interphase between the 1.4 M and 1.2 M sucrose layers. Buffer D was then added to a final volume of 4 mL, and the mixture was centrifuged again at 12,300× *g* for 30 min. The pellet was then resuspended in buffer D to a final total protein concentration of 1.2 mg/mL. All steps after removing the cells from the culture plates were performed on ice, and all centrifugation was carried out at 4 °C.

### 4.5. Transmission Electron Microscopy

Recombinant Atx(D49S) was conjugated to 5 nm N-hydroxysuccinimide (NHS)-activated gold nanoparticles (GNP) (Cytodiagnostics, Burlington, Canada), according to the manufacturer instructions, using conjugation kits (Cytodiagnostics, Burlington, Canada). Briefly, Atx(D49S) was vacuum dried and resuspended in resuspension buffer to a final Atx(D49S) concentration of 0.5 mg/mL. Reaction buffer was added to the Atx(D49S), and the mixture transferred to a vial containing lyophilised NHS-activated GNP and mixed immediately. After a 2-h incubation at room temperature, 10 µL quencher solution was added to the mixture, to stop the reaction. The mixture was then transferred to a Centricon protein concentrator with a 100-kDa cut-off (Merck Millipore, Burlington, MA, USA), centrifuged at 1000× *g* for 20 min, and then diluted with 0.5 mL PBS. The process of dilution and concentration was then repeated at least five times. The Atx(D49S)-GNP conjugate prepared in this way was stored at 4 °C until use. Lysozyme-GNP conjugate was prepared as described for the Atx(D49S)-GNP.

A total of two days after their plating, PC12 cells were incubated for 2 h in either the absence (control) or the presence of 1 µM Atx(D49S)-GNP in PBS at 37 °C under 5% (*v*/*v*) CO_2_. The cells were fixed with a mixture of 4% (*m*/*m*) formaldehyde and 2% (*m*/*m*) glutaraldehyde in 0.1 M cacodylate buffer (pH 7.4) for 2 h at room temperature. After fixing, the cells were rinsed overnight in 0.1 M cacodylate buffer. Post-fixation was carried out in 1% (*m*/*v*) OsO_4_ in 0.1 M cacodylate buffer for 2 h at 4 °C, followed by dehydration through graded ethanol concentrations and embedding in epoxy resin–glycid ether 100 (Serva Electrophoresis, Heidelberg, Germany). Some ultrathin sections from the control samples were counterstained with uranyl acetate and lead citrate, and all sections were examined under TEM (CM100; Royal Philips Electronics, Amsterdam, The Netherlands).

Mitochondria isolated from PC12 cells were incubated in either the absence (control) or the presence of 1 µM Atx(D49S)-GNP in buffer D for 1 h at 37 °C. After incubation, the mitochondria were centrifuged at 16,000× *g* for 30 min at 4 °C, washed with buffer D, and fixed with a mixture of 4% (*m*/*m*) formaldehyde and 2% (*m*/*m*) glutaraldehyde in 0.1 M cacodylate buffer (pH 7.4) for 2 h at 4 °C. Post-fixing, embedding in resin, preparation of ultrathin sections and their examination were carried out as described above.

### 4.6. Immunofluorescence Co-Localisation of Atx(D49S) in PC12 Cells

Recombinant Atx(D49S) was conjugated to Alexa Fluor 546 dye (Alexa Fluor^546^ protein labelling kit; Molecular Probes Life Technologies, USA), the reaction products separated using RP-HPLC, and the Alexa mono-derivative of Atx(D49S), ^546^Alexa-Atx(D49S), was further characterised as described by Oberčkal et al. (2015) [9]. The co-localisation study of ^546^Alexa-Atx(D49S) and CCOX-II in PC12 cells was performed as described by Šribar et al. (2019) [12].

## Data Availability

Data is contained within the article or supplementary material.

## Supplementary Materials

The following supporting information can be downloaded at: https://www.mdpi.com/article/10.3390/toxins14060375/s1, Figure S1: Biochemical characterization of recombinant enzymatically inactive ammodytoxin; Figure S2: Distribution of the lysozyme conjugated to gold nanoparticles (GNP) in PC12 cells using transmission electron microscopy (TEM).
