# Supplementary material for: The Phospholipase Activity of Ammodytoxin, a Prototype Snake Venom β-Neurotoxin, Is Not Obligatory for Cell Internalisation and Translocation to Mitochondria
Source: Toxins (Basel). 2022 May 28;14(6):375. doi: 10.3390/toxins14060375 (PMC9228470; doi:10.3390/toxins14060375)
Supplement: Supplementary file 1 [file toxins-14-00375-s001.zip › toxins-1721382-supplementary.pdf]

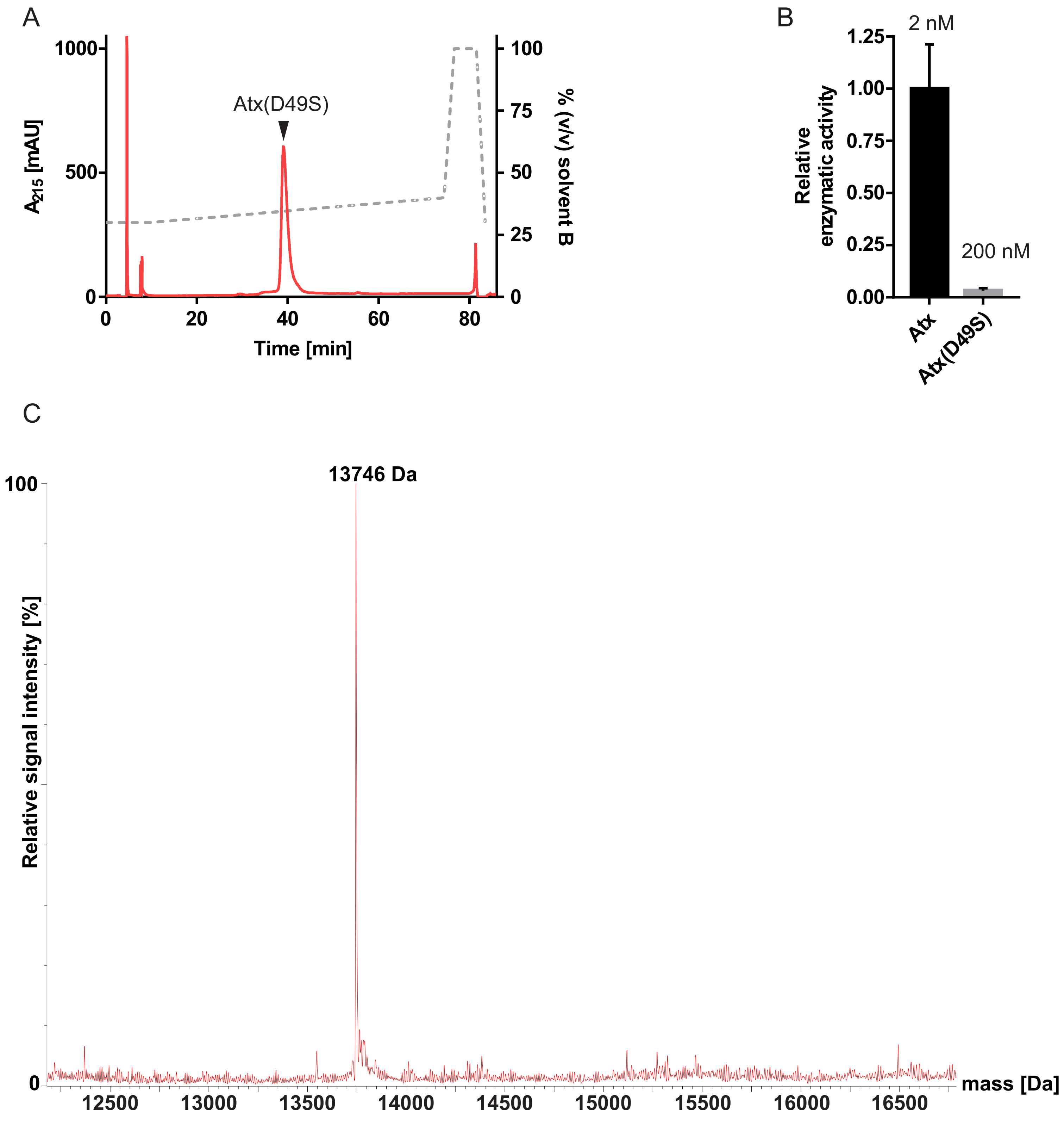

Figure S1. Biochemical characterization of Atx(D49S), recombinant enzymatically inactive mutant of ammodytoxin. **(A)** A reverse-phase HPLC chromatogram of the final purification step of Atx(D49S). Atx(D49S) eluted in a sharp, symmetrical peak. **(B)** Comparison of the enzymatic activity of the wild type Atx and Atx(D49S) on PyrPG as the substrate. The enzymatic activity of Atx(D49S) was negligible compared to activity of the wild type Atx. **(C)** The mass spectrum of Atx(D49S) preparation consisted of a single sharp peak at 13746 Da. The 0.76 Da deviation from the theoretical mass of Atx(D49S) is in the range of the experimental error. This demonstrated homogeneity of the preparation, and the correct folding of the Atx mutant. Methodological details are described under Materials and Methods, in section 4.2.

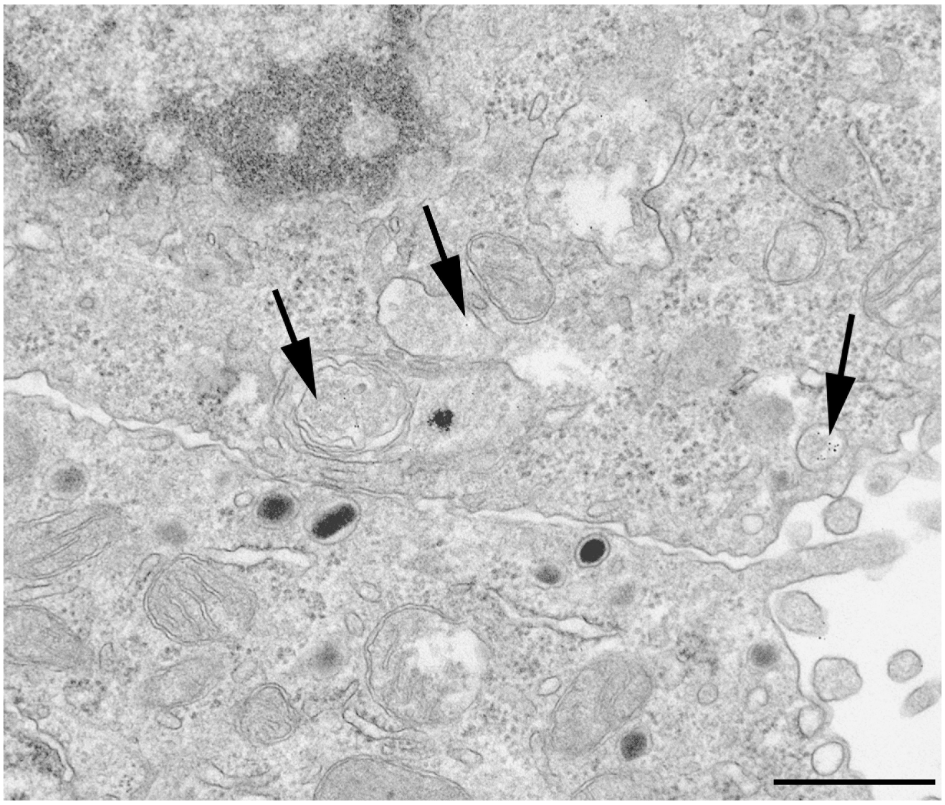

Figure S2. Distribution of the lysozyme conjugated to gold nanoparticles (GNP) in PC12 cells using transmission electron microscopy (TEM). Representative TEM image of PC12 cells incubated with lysozyme-GNP for 2 h shows electron-dense particles were present in endocytotic vesicles (black arrows), but not in the cytosol or endoplasmic reticulum as found for the enzymatically inactive mutant Atx(D49S) conjugated to GNP. Scale bar, 600 nm.
